# Cold-Renneted Milk Powders for Cheese Production: Impact of Casein/Whey Protein Ratio and Heat on the Gelling Behavior of Reconstituted Rennet Gels and on the Survival Rate of Integrated Lactic Acid Bacteria

**DOI:** 10.3390/foods10071606

**Published:** 2021-07-11

**Authors:** Malou Warncke, Sonja Keienburg, Ulrich Kulozik

**Affiliations:** Chair of Food and Bioprocess Engineering, TUM School of Life Sciences, Technical University of Munich, Weihenstephaner Berg 1, 85354 Freising, Germany; keienburgsonja@yahoo.de (S.K.); ulrich.kulozik@tum.de (U.K.)

**Keywords:** cheese manufacture, ultrafiltration, microfiltration, cold-renneting, spray drying, whey protein denaturation, milk protein concentrates, milk protein powders

## Abstract

The idea was to develop powders for fresh/hard cheese or quark production comprising milk proteins in optimal composition and functional properties for manufacturing each of those cheese types. The aim was to avoid whey protein drainage by their prior removal or by their heat-induced structural integration in the curd. The pre-renneted powders already contain additives such as starter cultures and calcium chloride to instantaneously form homogeneous curds upon reconstitution. The impact of the casein/whey protein ratio (86:14 by ultrafiltration and 98:2 by microfiltration) and upfront heat treatment (80 °C/30 min) on the gelling behavior of reconstituted rennet gels and on the survival rate of integrated *Lactobacillus paracasei* ssp. *paracasei* F19 was investigated. The assessment criteria for the rennet gelation were curd firming rate, gel strength, and whey drainage. Furthermore, the amount of integrated whey proteins and the resulting cheese yield were evaluated. It could be shown that heating had a positive effect on the viable cell count of the bacteria after spray drying and on the gelation behavior of the reconstituted ultrafiltration concentrates. The curd firming rate and the gel strength could be increased to higher values than the reconstituted microfiltration concentrate at 25% total solids.

## 1. Introduction

During cheese manufacturing, 70% of the starting amount of milk is converted to sweet whey, which is commonly purified to obtain the isolated whey proteins. In some parts of the world that lack infrastructure for whey processing, the whey must be discarded and then potentially creates environmental burden. A novel concept to avoid whey disposal studied in this work is powder-based cheese manufacturing with high yield due to an increased casein/whey protein ratio or the integration of the whey proteins in the cheese matrix.

To avoid losses of valuable whey proteins, heat treatment could be applied up to a certain degree to denature and structurally integrate the whey proteins in the cheese curd [1]. However, heat treatment prior to renneting affects the rennetability of the casein micelles due to whey proteins blocking the casein micelle surface. Steffl [2,3] reported on an impaired rennet gelation if β-lactoglobulin denaturation exceeded 60%. Then, the binding sites (the κ-casein) are partly occupied and not accessible for the rennet anymore. In contrast to Steffl [2,3], Anema et al. [4] found that the observed retarded rennet gel formation did not depend on whether denatured whey proteins were associated with the casein micelles or self-aggregated in the serum phase. Thus, these authors assumed that these complexes inhibit further aggregation and, therefore, retard the gelation. 

However, these detrimental effects of present whey protein aggregates on rennet gelation can be circumvented by ultrafiltration (UF) concentrating or increasing the casein/whey protein ratio, i.e., by reducing the whey protein content by microfiltration (MF) prior to heat treatment. Schreiber [5,6] reported that at casein concentrations above 8% the gel strength of heated UF concentrates was higher than the gel strength of pasteurized skim milk, even if all whey proteins were denatured. The integration of denatured whey proteins into the cheese matrix seems therefore possible if the casein concentration is high enough. Bulca [7] found that skim milk with casein/whey protein ratios of 3.4:0.01% or 6.4:0.65% heat-treated at 140 °C for 10 s gelled as fast as pasteurized skim milk and showed the same gel firmness.

Based on these reports the idea of this study was to develop powders with the optimal composition for wheyless manufacturing of rennet-based cheese types. The powder for fresh/hard cheese should have a high casein and a low whey protein concentration. For this, the casein concentration was increased, and the whey proteins were removed at the same time by MF in diafiltration mode. Considering powder for quark production, it should have a high total protein concentration, whereby the whey proteins are denatured and bound to the casein micelles. This can be achieved by heating UF retentates. The main advantages are, on the one hand, the increased cheese yield due to the higher protein concentration and, on the other hand, the omitted sweet whey collection and purification for the consumer. Furthermore, the powders already have the optimal composition making the prior alteration of protein concentration and composition of the vat milk needless. The aim was to develop pre-renneted milk protein concentrate powders containing starter cultures and calcium chloride, ready to form homogeneous gel matrices upon rehydration. 

A conceptionally similar study was conducted by Würth et al. [8], who developed a spray drying process to encapsulate probiotics in milk matrices with the classic rennet gelation process for cheese manufacture. Skim milk containing *Lactobacillus paracasei* ssp. *paracasei* F19 was cold-renneted at 4 °C prior to spray drying, where aggregation of the para-casein micelles does not occur [9,10]. This so-called ‘capsule precursor powder’ formed the final water-insoluble hydrogel capsules upon rehydration at temperatures above 16 °C. 

The fact that milk matrices are suitable for integrating bacteria in a protective environment was also shown by Khem et al. [11]. They reported that whey protein isolates (WPI) and skim milk have a higher protective effect on *Lactobacillus plantarum* than carbohydrates such as lactose or trehalose during drying. The reason was the crust formation in the early drying stage. This creates a shell and, thus, prevents the droplet from overheating at the later drying stages. Moreover, the calcium present in milk was postulated to increase the intrinsic heat resistance of the lactic acid bacteria. Desmond et al. [12] reported that adding 0.3 M sodium chloride to a suspension of reconstituted skim milk (20% (*w*/*v*)) and *Lactobacillus paracasei* resulted in a high degree of cross-protection for the bacteria against heat stress during spray drying. 

Khem et al. [13] investigated the effect of denatured whey proteins on the survival of *Lactobacillus plantarum* spray-dried in WPI, lactose, or WPI/lactose mixtures. The significantly higher survival rates in WPI compared to lactose or WPI/lactose mixtures led them to assume that the whey proteins have a protective effect on the bacteria. These authors thought that the whey proteins unfold during spray drying at an outlet temperature of approximately 70 °C and that these interact hydrophobically with the hydrophobic bacteria causing aggregate formation. As a result, the microorganisms embedded in the capsule are protected against inactivation during spray drying. Based on these results it would be obvious that heated WPI solutions may have a better protective effect on bacterial cells because higher degrees of whey protein denaturation could be achieved. Moreover, Picot and Lacroix [14] encapsulated *Bifidobacterium breve* in heated WPI solutions (80 °C/30 min). They achieved survival rates of 26% (corresponding to 10^9^ cfu mL^−1^, cfu = colony forming units) when spray drying with an air inlet and outlet temperature of 160 °C and 80 °C, respectively. 

Based on the studies mentioned above, it was hypothesized that (1) cold-renneted skim milk UF and MF powders result in a homogenous gel matrix upon rehydration at temperatures below 16 °C; (2) UF concentration allows to integrate a high amount of denatured whey proteins without impairing the renneting properties; (3) the survival rate in the heated concentrates should be higher than in the unheated ones due to the protective effect of the whey protein aggregates.

The impact of the casein/whey protein ratio (86:14 and 98:2) and heat treatment (80 °C/30 min) on the gelling behavior of reconstituted rennet gels (meaning the reconstituted, gelled concentrates) and on the survival rate of integrated *Lactobacillus paracasei* ssp. *paracasei* F19 was investigated. The assessment criteria for the rennet gelation were curd firming rate, gel strength, and whey drainage. In addition, the amount of integrated whey proteins and the resulting cheese yield were evaluated. Furthermore, the viable cell count was determined after spray drying and during powder storage at 4, 10, and 20 °C during 103 days of storage. 

This study was designed to form the base for ‘instant’ cheese production based of pre-renneted powders containing a high number of viable cells and all required ingredients forming cheese, but without whey protein drainage. A powder with a high storage stability for transport across long distances could be possible, and the lactobacilli would be dormant due to lacking water, but rapidly activated upon rehydration.

## 2. Materials and Methods

### 2.1. Cold-Renneted Milk Protein Powder Production

**Figure 1:**
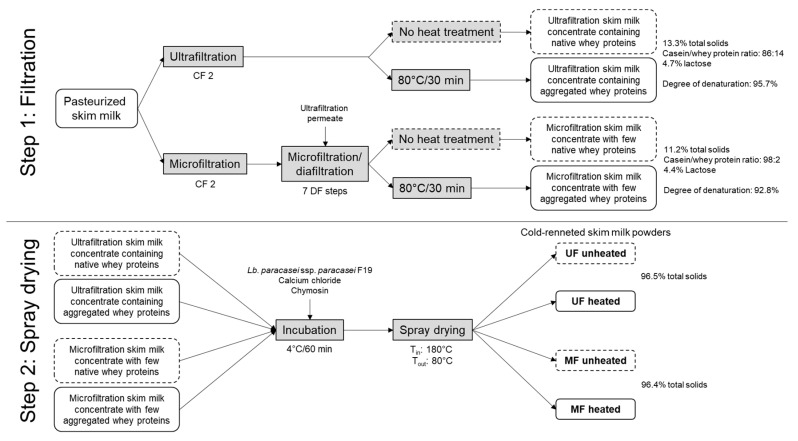
Processing scheme for cold-renneted skim milk powder production. CF: concentration factor; DF: diafiltration; UF: ultrafiltration; MF: microfiltration.

In this study, four cold-renneted skim milk powders varying in casein/whey protein ratio and whey protein denaturation were produced. Figure 1 shows the related process scheme.

Pasteurized skim milk (74 °C/28 s, Molkerei Weihenstephan GmbH & Co. KG, Freising, Freising, Germany) was ultrafiltered (UF) with a polymeric spiral-wound membrane (10 kDa, DSS Silkeborg AS, Silkeborg, Denmark) and microfiltered (MF) with a ceramic module (0.14 µm, TAMI Industries, Nyons, France). During UF, the total protein concentration was increased from 3.8 to 6.9%. During MF and diafiltration, the casein concentration was increased from 3.3 to 5.5%, whereas the whey protein content decreased from 0.5 to 0.1%. UF permeate was used as diafiltration medium to keep the natural milk milieu. Hence, the lactose concentration was constant between 4.7 and 4.4% in the UF and MF concentrates, respectively. The filtration temperature was 50.0 ± 1.0 °C. Casein and whey protein content (according to Dumpler et al. [15]) and lactose content (according to Schmitz-Schug et al. [16] were determined by reversed-phase high-performance liquid chromatography (RP-HPLC).

Since the concept was to integrate the whey proteins in the cheese matrix by denaturation, each concentrate was split in two halves (see Figure 1). One half remained unheated, whereas the other half was heated at 80 °C for 30 min under steady stirring in a water bath to induce whey protein aggregation. The degree of whey protein denaturation was calculated according to Warncke et al. [17]. The final degrees of denaturation were 95.7 ± 0.7% for the UF concentrate and 92.8 ± 0.5% for the MF concentrate. All samples were stored at 4.0 ± 1.0 °C overnight. 

Before spray drying, the probiotic strain *Lactobacillus paracasei* ssp. *paracasei* F19 (in the following *Lb. paracasei*) (Chr. Hansen A/S, Hørsholm, Denmark), calcium chloride (Effinger Klaus Käser- & Imkerbedarf, Sonthofen, Germany), and rennet (CHY-MAX^®^ M 1000, Chr. Hansen A/S, Hørsholm, Denmark) with an enzyme activity of 1000 IMCU L^−1^ (international milk clotting units) were added to the four concentrates. The concentrations were 10.56 g culture (corresponds to 2.9 × 10^9^ cfu mL^−1^) and 0.15 mL calcium chloride per kg protein concentrate. The chymosin was added with a concentration of 2.303 µL per gram casein. The concentrates were gently stirred for 10 min to distribute the ingredients homogeneously before resting for 50 min to give the chymosin time for hydrolysis of the κ-casein.

Spray drying was performed by a NIRO Atomizer (GEA Group, Düsseldorf, Germany). The inlet air temperature was 180.0 ± 1.9 °C, and the outlet air temperature was 80.0 ± 2.2 °C to achieve a moisture content below 4% (*w*/*w*) for a good powder quality [18] and powder storage stability [19,20]. The feed flow rate was 0.3 ± 0.03°L min^−1^, and the disk rotation speed was 15,000 rpm. The double-walled feed tank was tempered to 4.0 ± 0.5 °C to avoid a premature aggregation of the para-casein micelles. 

The powders were stored in aluminum compound foil bags to avoid oxygen migration through the packaging material and to prevent the powders from UV radiation. The storage temperatures were 4, 10, and 20 °C to investigate the impact of the storage temperature on the viable cell count of the bacteria. The moisture content and the a_w_-value of the powders were determined by the CEM Smart6 Turbo (CEM GmbH, Kamp-Lintfort, Germany) and the HygroLab C1 (Rotronic Messgeräte GmbH, Ettlingen, Germany), respectively. Each powder was produced twice.

### 2.2. Viable Cell Count of Lactobacillus Paracasei SSP. Paracasei F19

Before determining the viable cell count in the powders, the bacteria were isolated enzymatically according to Heidebach [21]. For this, 1 g powder was mixed with 1 mL phosphate buffer (pH 7), 1 mL of the protease from *Lactobacillus amyloliquefaciens* ssp. *amyloliquefaciens* (Sigma-Aldrich, St. Louis, MO, USA), and 7 mL deionized water (10 °C) in a 50 mL falcon tube. After 65 min in the New Brunswick Innova 42/42R Incubator (Eppendorf AG, Hamburg, Germany) at 40 °C and shaking at 110 rpm, the powders were dissolved, and the rennet gel, which built inevitably upon rehydration, was disintegrated. 

The colony-forming units (cfu) were determined using a serial dilution in Ringer’s solution (Merck KGaA, Darmstadt, Germany) up to 10^−7^, which was subsequently plated on MRS agar. The plates were incubated anaerobically at 37 °C for 48 h. Plates with up to 300 cfu were used for calculating the cfu per mL according to the established microbiological standard method [22]. All serial dilutions were performed in duplicate.

The survival rate was calculated by Equation (1), where N_0_ is the viable cell count (cfu mL^−1^) after spray drying (corresponds to day 0), and N is the viable cell count after x days of storage.

(1) $$Survival\;rate\;\left(\%\right)=\left(\frac{N}{N_{0}}\right)\cdot 100\%$$

### 2.3. Surface Hydrophobicity of Lactobacillus Paracasei SSP. Paracasei F19 under Heat Stress

The change in surface hydrophobicity of *Lb. paracasei* upon heating was tested according to Khem et al. [13]. For this, 15.0 ± 0.02 g of the *Lb. paracasei* culture was mixed with 50 mL of 10 °C deionized water in duplicate. To induce heat stress, one suspension was warmed to 45 °C under steady stirring in a water bath for 10 min according to Haddaji et al. [23]. Afterward, it was cooled on ice for 5 min. 

Twenty-five milliliters of the unheated and heated suspension was mixed with 5 mL hexadecane in a falcon tube. After 15 min of incubation, the supernatant was clear (bacteria were hydrophilic) or milky white (bacteria were hydrophobic and solved in the hydrophobic solvent). 

### 2.4. Curd Firming Rate and Strength of Reconstituted Rennet Gels

To evaluate the impact of the casein/whey protein ratio and denatured whey proteins on the rennet gelation properties of the cold-renneted reconstituted concentrates, the curd firming rate and the gel strength were determined. 

Before the measurements, the powders were redispersed in 10 °C deionized water to 10 and 25% (*w*/*w*) total solids. To avoid a premature aggregation, the beaker was constantly on ice. After homogenizing with the Ultra-Turrax (IKA-Werke GmbH & CO. KG, Staufen, Germany) at 10,000 min^−1^ for 5 min, the samples were stored at 4 °C overnight under steady stirring. Before the measurements, the samples were homogenized again under the same conditions to ensure full powder solubilization [24,25].

For both measurements, the MCR 302 rheometer (Anton Paar GmbH, Graz, Austria) equipped with the cone/plate geometry (d = 50 mm, 2°) was used. The sample volume was 1.15 mL. Oscillation at a constant deformation (0.01%) and frequency (1 Hz) was applied for 7.2 min, whereby the sample formed a gel. At the same time, the temperature increased linearly from 4 to 40 °C. Having reached 40 °C, the temperature was held for 30 min. The curd firming rate was defined as the increase in the storage modulus G’ within 300 s. The curd firming rate was calculated by Equation (2), where 0 s is that time, where the temperature reached 40 °C.

(2) $$Curd\;firming\;rate\;\left(\mathrm{Pa}\;s^{-1}\right)=\frac{G_{300\;s}^{\prime }-G_{0\;s}^{\prime }}{300\;s}$$

For measuring the gel strength, an amplitude sweep was applied. First, the samples were sheared at 500 s^−1^ for 1 min at 4 °C to give them time to equilibrate. After resting for 30 s, the temperature increased to 40 °C within 10 min to induce rennet gel formation. After reaching and holding 40.0 ± 0.02 °C for another 10 min, the oscillating amplitude sweep (logarithmic ramp from 0.01 to 100% deformation at a constant frequency of 1 Hz) was applied to determine the gel strength in the linear viscoelastic region (LVR) (corresponds to 0.01–0.05% deformation). All measurements were performed in duplicate.

### 2.5. Whey Drainage, Whey Protein Integration in the Curd, and Cheese Yield

The whey drainage, which occurs upon curd cutting and pressing, is an important criterion in cheese manufacturing, which defines the dry matter and, therefore, the hardness of the final cheese. For whey drainage determination, 40.0 ± 0.5 g of the reconstituted, homogenized sample was weighed in a 50 mL falcon tube. After 1 h of incubation at 40 °C, the samples were centrifuged at 4000× *g* for 45 min at 20 °C. The supernatant was immediately weighed, and the whey drainage was calculated by Equation (3), where $m_{serum}$ (g) is the serum mass and $m_{0}$ (g) the mass of the whole sample.

(3) $$Whey\;drainage\;\left(\%\right)=\frac{m_{serum}}{m_{0}}\cdot 100\%$$

To evaluate the impact of heat on the whey protein integration in the cheese matrix, the whey protein concentration in the sweet whey was analyzed by RP-HPLC. The cheese yield was calculated as follows (Equation (4)), where m_0_ is the mass of the whole sample.

(4) $$Cheese\;yield\;\left(\mathrm{kg}\;100\;{\mathrm{kg}}^{-1}\right)=\frac{m_{curd}}{m_{0}}\cdot 100$$

The highest cheese yield was expected for the heated UF sample because the number of proteins, which can structurally contribute to the gel network, was highest. Both analyses were performed in duplicate.

### 2.6. Statistical Analysis of Data

Origin 2021 (OriginLab Corporation, Northampton, MA, USA) was used to plot graphs, and RStudio, Inc., 2019 (version 1.2.5033, Boston, MA, USA) was used for statistical analysis. Statistical significances (*p* ≤ 0.05) were evaluated using one-way analysis of variance (ANOVA) combined with Tukey’s HSD post hoc test. Significant differences were calculated for values of different variables measured in two independent powders.

## 3. Results and Discussion

### 3.1. Survival of Lactobacillus Paracasei SSP. Paracasei F19 after Spray Drying and during Powder Storage

**Figure 2:**
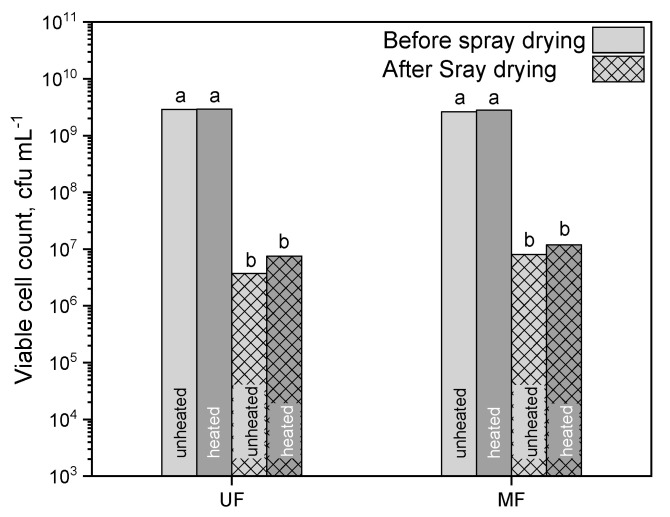
Viable cell count before and after spray drying of UF and MF. The standard deviations of all samples before spray drying were ±5 × 10^4^ cfu mL^−1^ and after spray drying 2 × 10^3^ cfu mL^−1^ (presented, but not visible due to scale). Letters indicate the statistical significance at *p* ≤ 0.05.

Figure 2 illustrates the viable cell count of the ultrafiltration (UF) and microfiltration (MF) samples before and after spray drying. Cell counts N_0_ before spray drying of the unheated and heated UF and MF samples were not significantly different (*p* = 1.00) with ~3 × 10^9^ cfu mL^−1^. Spray drying caused a significant reduction in the viable cell count by ~2–2.5 log levels to 3.7 × 10^6^–1.2 × 10^7^ cfu mL^−1^ in all four samples (*p* ≤ 0.001). This means a survival rate of 13 and 31% in the unheated UF and MF powders, respectively. This is in the range reported by Teanpaisan et al. [19], who spray-dried *Lb. paracasei* in reconstituted skim milk with 20% total solids at 170/80 °C air inlet/outlet temperature. 

The differences between the UF and MF concentrates can be attributed to the differences in total solid content of the concentrates before spray drying. Due to whey protein depletion, the total solid content in the MF concentrate was 11.2%, whereas the UF concentrate contained 13.3% total solids. The higher the total solid content, the faster a droplet reaches and exceeds the wet bulb temperature. Consequently, the heat stress for bacteria at a constant residence time is higher [26]. However, the cell count was still above 3 × 10^6^ cfu g^−1^. The viable cell count after spray drying can be increased by inoculating a higher cell number, by decreasing the total solid content of the feed solution, or by decreasing the drying temperature [26]. However, the moisture content needs to be considered as it should not exceed 4% (*w*/*w*) for a good powder quality [18] and powder storage stability [19,20].

As hypothesized, the heated samples contained more viable cells after spray drying than the unheated ones. The survival rate in the heated UF powder was 25%, whereas the survival rate in the heated MF powder was 43%. It could be assumed that the heated milk constituents have a protective effect caused by hydrophobic interactions on the bacteria, as already shown by Khem et al. [13].

The surface hydrophobicity of the milk proteins can be altered by heating or renneting. During denaturation, the whey proteins unfold and expose their hydrophobic regions. They can either bind to κ-casein on the casein micelles’ surfaces, to serum κ-casein, or build disulfide-bonded and hydrophobically associated serum aggregates [27]. These serum aggregates and casein/whey protein complexes bear a significantly higher surface hydrophobicity than unheated casein micelles [28,29]. Another way to turn the casein micelles hydrophobic is renneting. Rennet cleaves the hydrophilic κ-casein on the surface into para-κ-casein and casein macropeptide. Consequently, the casein micelles become more hydrophobic. This raised the question whether *Lb. paracasei* in our study turned hydrophobic during spray drying as well, which would confirm the hypothesis of Khem et al. [13]. The hydrophobicity test indicated the change from a hydrophilic to a hydrophobic surface of the bacterial cells when heat stressed, just as the bacteria experience it in the spray dryer. Due to the prior heating, more denatured—and hence, hydrophobic—whey proteins are available in the heated UF and MF samples, which potentially act as a protective shield for the bacteria against overheating. Consequently, the viable cell count in the heated samples was higher after spray drying. 

**Figure 3:**
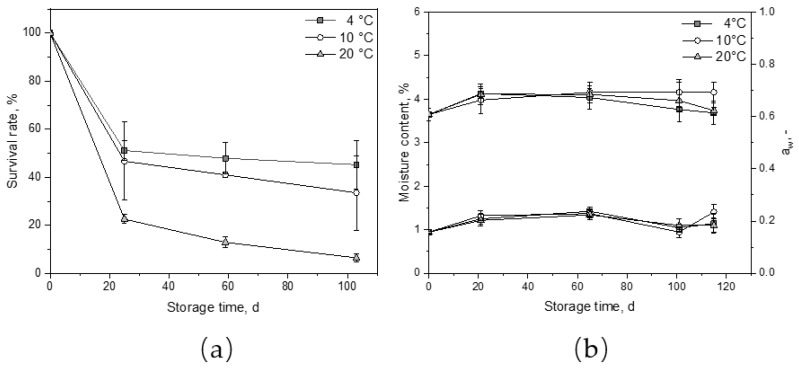
Survival rate of *Lactobacillus paracasei* ssp. *paracasei* F19 (**a**) and moisture content and a_w_ (**b**) as a function of storage time.

The survival rates of the lactobacilli cells during powder storage at different temperatures were further investigated. Figure 3a shows the survival rate of *Lb. paracasei* during 103 days of storage at 4, 10, and 20 °C. Day 0 corresponds to the viable cell count after spray drying. The powder composition as well as the state (unheated/heated) had no significant impact on the survival rate during storage (*p* = 0.86). The highest survival rates were achieved at 4 °C with 51% after 25 days and 45% after 103 days of storage. The survival rates of the cells in the powders stored at 10 °C showed a slightly lower survival rate with 46% after the first 60 days of storage. Storage at 20 °C yielded the lowest survival rate with a loss of almost 80% within the first 25 days. After 103 days, only 6.5% of *Lb. paracasei* were still vital, which confirms results reported in [19,20,30,31,32]. The reason for this could be the damaged lipid cell membrane and the resulting lipid oxidation [30]. The powders were simultaneously analyzed regarding their moisture content and a_w_-value. Figure 3b shows the change in moisture content and a_w_-value over the powder storage period. The moisture content increased only slightly from 3.6 to 4.1% and the a_w_ from 0.16 to 0.2 in all powders within the first 21 days of storage. The a_w_ was around 0.2, which was in the suitable range (max. 0.65) for bacterial survival in milk-based powders [33]. This allows the losses in survival rate to be attributed to the temperature only, which seemed to be the main factor affecting the survival rate of the bacteria during powder storage. Nevertheless, even after 103 days of storage at 20 °C, the viable cell count was still above 10^6^ cfu g^−1^.

In summary, it can be said that the storage and transport of the powders at 20 °C is possible and that a viable cell count of 10^6^ cfu g^−1^ can be ensured when inoculating the milk concentrates with N_0_ = 2.9 × 10^9^ cfu mL^−1^. 

### 3.2. Rennet Gelation Behavior of Reconstituted Cold-Renneted Skim Milk Concentrates

In the following chapter, the curd firming rate, gel strength, whey drainage, whey protein integration, and cheese yield of the reconstituted cold-renneted skim milk concentrates are presented.

#### 3.2.1. Curd Firming Rate

**Figure 4:**
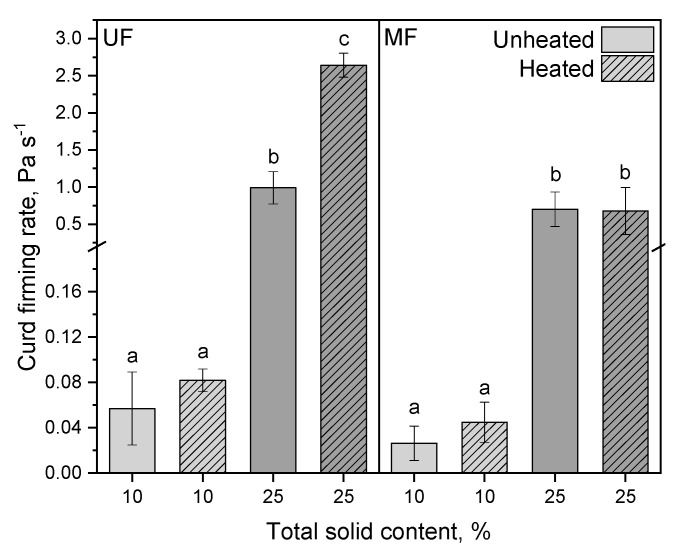
Curd firming rate of unheated and heated reconstituted UF and MF rennet gels with 10 and 25% total solid content. Letters indicate the statistical significance at *p* ≤ 0.05.

The curd firming rate is defined as the increase in the storage modulus G’ within 300 s. Figure 4 illustrates the curd firming rate of unheated and heated UF and MF rennet gels with 10 and 25% (*w*/*w*) total solids.

Heated UF concentrate showed a higher curd firming rate compared to the unheated counterpart as it increased from 0.06 to 0.08 Pa s^−1^ at 10% total solids. At 25% total solids the curd firming rate significantly increased from 0.99 to 2.64 Pa s^−1^ (*p* = 0.002) (Figure 4). This indicates that the first phase of the renneting process, the enzymatic cleavage of κ-casein by hydrolysis at 4 °C prior to spray drying, was not impaired by whey protein denaturation, and that aggregation occurred upon powder rehydration at elevated temperature. Anema et al. [4] and Mollé et al. [34] showed that chymosin is able to cleave the κ-casein in soluble heat-induced κ-casein/whey protein aggregates. However, our results are in contrast to those reported by Anema et al. [4], where heating skim milk at 90 °C for 5–30 min resulted in a retarded rennet gelation process, and Steffl [2,3], who reported that above a whey protein denaturation degree of 60%, milk remains liquid. Anema et al. [4] observed a retarded gelation after heating (90 °C/15 min) as well and explained this with the disturbance of the denatured whey proteins in the aggregation process—independent of whether the whey proteins are complexed with the casein micelles or serum κ-casein. In this study, milk concentrates with either a higher total protein concentration or a higher casein/whey protein ratio compared to skim milk were used. Bulca et al. [35], Waungana et al. [36], and Schreiber [5,6] already showed that concentrating as well as increasing the casein/whey protein ratio compensate these longer gelation times or weaker gels of ultrahigh temperature-treated skim milk, so that rennet gelation is still possible even at high degrees of whey protein denaturation. It can be assumed that the increased hydrophobicity of the casein/whey protein complexes may not only protect the bacteria from cell death during spray drying but could also be the reason for the higher curd firming rate in heated UF samples. This would be in accordance with the results found for the MF concentrates, where the unheated and heated samples behaved similarly, especially at 25% total solids. At 10%, the curd firming rate remained around 0.03 Pa s^−1^ and at 25% around 0.69 Pa s^−1^. Here, the whey protein concentration was much lower, less casein/whey protein aggregates formed upon heating, and consequently, the hydrophobicity of the casein micelles was only altered by the rennet but not increased further by attached whey proteins. 

#### 3.2.2. Gel Strength and Whey Drainage

**Figure 5:**
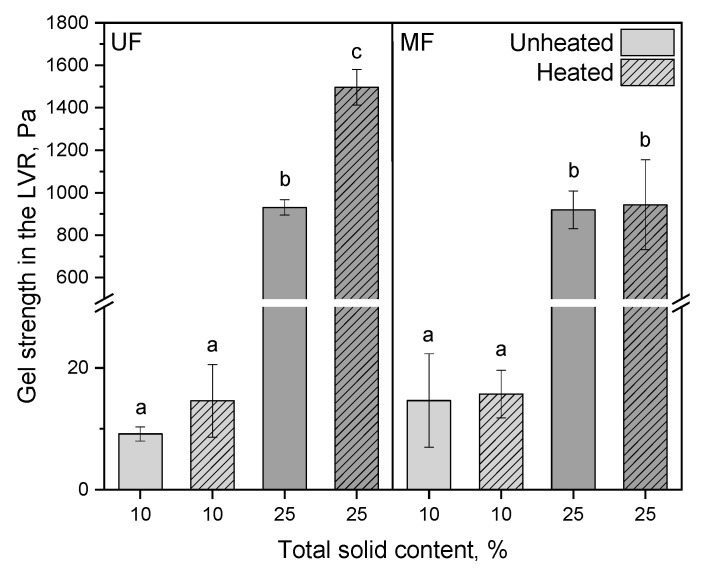
Gel strength in the linear viscoelastic region (LVR) of unheated and heated reconstituted UF and MF rennet gels with 10 and 25% total solid content. Letters indicate the statistical significance at *p* ≤ 0.05.

The gel strength in the linear viscoelastic region (LVR) was determined by rheometry. This method provides insights regarding gel firmness without destructing the gel network, as in penetration measurements. The gel strength displays the curd firmness after full formation before cutting. The same trends were observed for the gel strength in the LVR as for the curd firming rate. Figure 5 illustrates that a high curd firming rate went hand-in-hand with a high gel strength. 

The gel strengths of the UF and MF samples at 10% total solids were ~15 Pa, whereas the gel strength at 25% total solids were with 930 Pa significantly higher (*p* ≤ 0.001) and increased up to 1,496 Pa when reconstituting heated UF concentrate powder. The higher gel strength at 25% total solids compared to 10% can be explained with the higher casein and colloidal calcium concentration (Figure 5). The more concentrated para-casein micelles are, the faster they contact each other and aggregate by calcium bridges. The strength of the network depends on the amount of involved casein micelles and, hence, the amount of calcium bridges. The corresponding loss moduli for the unheated and heated UF samples at 10% total solids were 3.1 ± 0.4 Pa and 4.5 ± 1.8 Pa, respectively, and at 25% total solids they were 247.6 ± 15.3 Pa and 405.4 ± 32.9 Pa, respectively. The corresponding loss moduli for the unheated and heated MF samples at 10% total solids were 4.3 ± 2.1 Pa and 4.8 ± 1.0 Pa, respectively, and at 25% total solids they were 229.9 ± 15.3 Pa and 254.5 ± 63.2 Pa, respectively.

Our results demonstrate that a rennet gel made of heated, cold-renneted UF concentrate powder shows a significantly higher gel strength than the gel made from the unheated powder at 25% total solids as the gel strength increased from 931.1 to 1496.5 Pa (*p* = 0.02). Our results contrast with the results from the literature showing softer and weaker gels made from heated skim milk [2,4,37]. However, our results can hardly be directly compared to these studies by two reasons: first, we used UF and MF concentrates with a higher total protein concentration and/or a higher casein/whey protein ratio compared to skim milk; and second, in our process, hydrolysis and aggregation ran separately and not simultaneously, as in the common renneting process. Our results also indicate that cold-renneting for 60 min at 4 °C allowed the rennet to hydrolyze enough κ-casein in the heated concentrates so that the subsequent aggregation upon rehydration was not impaired. Having separated hydrolysis and aggregation, our study allows to conclude that casein aggregation is not disturbed by denatured whey proteins. It can be assumed that a partly too fast aggregation—rather than the whey protein aggregates—hinders the rennet to hydrolyze all present κ-caseins, leading to an incomplete hydrolysis and an insufficient aggregation in the common rennet gelation process. Singh and Waungana [37] assumed that when heating UF concentrates, the nature of κ-casein/whey protein aggregates and the way in which casein micelles and whey proteins complex are different to that in heat-treated normal milk. These authors pointed out that the altered milk salt equilibrium in UF concentrates could also affect heat-induced changes in proteins and minerals.

**Figure 6:**
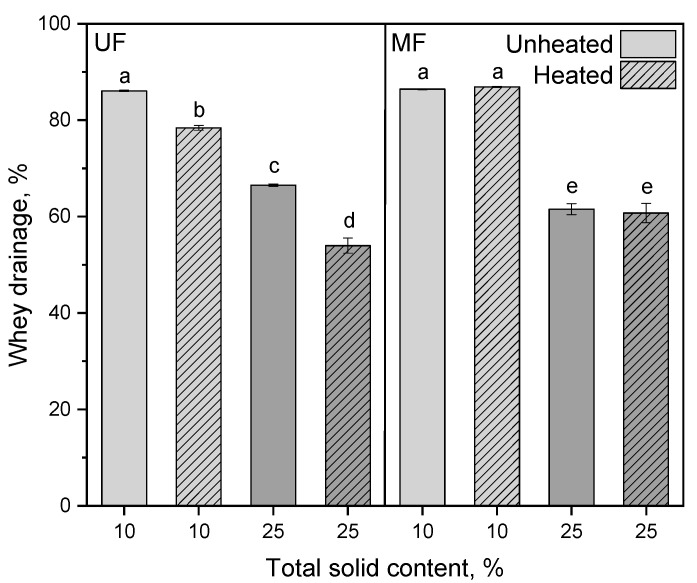
Whey drainage after incubation (t = 1 h, ϑ = 40 °C) and centrifugation at 4000× *g* (t = 45 min, ϑ = 20 °C) of unheated and heated reconstituted UF and MF rennet gels with 10 and 25% total solid content. Letters indicate the statistical significance at *p* ≤ 0.05.

The higher gel strength of the heated UF samples can be also explained by the higher serum holding capacity and consequently lower whey drainage. Figure 6 shows the whey drainage of the unheated and heated UF and MF samples at 10 and 25% total solids. As expected, a higher total solid content resulted in a lower whey drainage due to the increased solid/liquid ratio (78–86% at 10% total solids compared to 53–66% at 25% total solids). Furthermore, the heated UF samples showed a significantly lower whey drainage (*p* ≤ 0.001) than the unheated ones. In contrast to that, the MF samples did not show any significant differences (*p* = 0.99) between heated and unheated at the same total solid content.

After aggregation of the para-casein micelles, the gel network shrinks, which intensifies the whey drainage. Casein micelles can be considered as round-shaped spheres allowing a close contraction of the network. On the contrary, irregularly shaped whey protein aggregates and casein/whey protein complexes of different sizes, which form upon heating, hinder the network sterically from a close contraction. As a result, more whey retains in the pores, and the whey drainage decreases [37].

#### 3.2.3. Whey Protein Integration and Cheese Yield of Reconstituted Rennet Gels

**Figure 7:**
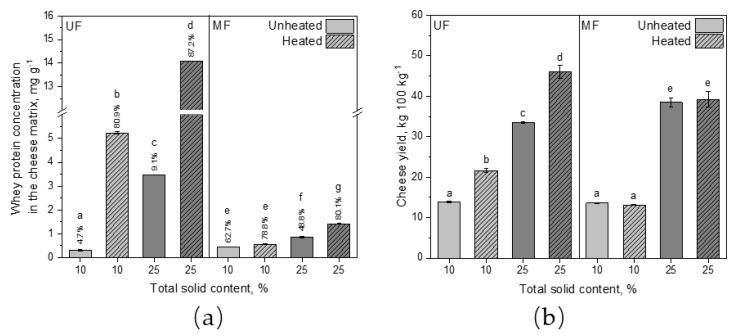
Whey protein concentration in the cheese matrix (**a**) and cheese yield (**b**) after incubation (t = 1 h, ϑ = 40 °C) and centrifugation at 4000× *g* (t = 45 min, ϑ = 20 °C) of unheated and heated reconstituted UF and MF rennet gels with 10 and 25% total solid content. Letters indicate the statistical significance at *p* ≤ 0.05.

The whey protein integration and the cheese yield of heated reconstituted UF and MF rennet gels compared to their unheated counterparts were further investigated. Figure 7a illustrates the whey protein concentration in the cheese matrix of unheated and heated UF and MF samples at 10 and 25% total solids. Heating allowed to integrate 81% of the whey proteins at 10% total solids and 87% at 25% total solids. In both cases this increase was significant (*p* ≤ 0.001). Steffl [2] also observed a 21% whey protein integration increase in heated cheese milk with 65% denatured whey proteins compared to unheated milk. On the contrary, only 4.7% and 9.1%, respectively, of the whey proteins were retained in the gel network without heating. This is in accordance with Warncke and Kulozik [25] who observed a ~5% whey protein retention in the cheese matrix of renneted MC88- and MPC85-enriched skim milk as well. Since the whey protein concentration in the MF samples was lower, the total amount of whey proteins in the cheese matrix was lower. However, up to 80% of the available whey proteins could be integrated as well. Interestingly, already 63% of the whey proteins in the unheated MF samples at 10% total solids and 49% at 25% total solids were integrated in the matrix. Heating did not significantly increase the whey protein concentration in the cheese matrix (*p* = 0.17) of the MF samples at 10% total solids, but it was significant at 25% (*p* ≤ 0.001). Now the question was whether the heat treatment of MF retentates has an impact on the cheese yield (Figure 7b). 

Heating MF concentrates had no beneficial effect on the cheese yield, as it remained around 13 kg 100 kg^−1^ at 10% total solids and around 39 kg 100 kg^−1^ at 25% total solids. Independent of whether the MF concentrate was heat-treated before spray drying or not, the cheese yield as well as the rennet gelation properties remained the same (*p* = 0.99). In contrast to that, the cheese yield of the UF samples could be increased significantly by heat treatment (*p* ≤ 0.001). At 10% total solids the cheese yield increased by 54% to 22 kg 100 kg^−1^ and at 25% by 37%. With 46 kg 100 kg^−1^ the heated UF sample with 25% total solids resulted in the highest cheese yield of all samples. The reason is a higher amount of integrated whey proteins as well as a higher serum retention in the pores [1,38]. The unheated and heated MF samples (25% total solids) were slightly lower with a cheese yield of 39 kg 100 kg^−1^ but still higher than the unheated UF sample at the same total solid content. Steffl [2] also reported on a 25% cheese yield increase in heated cheese milk with 65% whey protein denaturation. However, with increasing whey protein denaturation the yield increase decreased due to the soft curd texture resulting in a high amount of cheese dust, which gets lost during whey drainage [2]. This can be avoided when using heated concentrates since the gel strength was increased by heating, as shown in Figure 5.

## 4. Conclusions

This study demonstrates that the casein/whey protein ratio and heat affect the gelation behavior of reconstituted rennet gels and the survival rate of integrated *Lactobacillus paracasei* ssp. *paracasei* F19. Ultrafiltration (UF) and microfiltration (MF) concentrates were cold-renneted and mixed with *Lb. paracasei* and calcium chloride before spray drying. Aggregation occurred upon powder rehydration resulting in gel matrices with 10 and 25% total solids.

Based on the data obtained, the hypotheses could be confirmed in that (1) cold-renneted skim milk UF and MF powders result in a homogenous gel matrix upon rehydration at temperatures below 16 °C, when they got homogenized; (2) concentrating allows to integrate up to 87.2% of the denatured whey protein without impairing the renneting properties due to the sufficient amount of colloidal calcium and calcium bridges; (3) the survival rate in the heated concentrates was higher than in the unheated ones due to the more extensive whey protein denaturation and the probably more intense hydrophobic interactions.

The higher serum holding capacity of the UF curd makes this powder suitable for cheese types with higher moisture contents. For cheeses with a lower moisture content, the MF powders can be recommended due to their tightly meshed gel network and better whey drainage. MF concentrates have a high casein/whey protein ratio anyway; therefore, heating can be omitted regarding the rennet gelation properties. This process allowed to reduce the whey protein drainage by removal or integration without impairing the renneting properties, whereby the cheese yield can be increased. Heating is an appropriate tool for pre-treatment regarding powder functionalization, while increasing the number of viable integrated bacterial cells. This can be used, for example, in developing functional dairy foods containing active probiotics. If the amount of rehydration water could be chosen such that the final dry matter of a respective type of cheese would be reached, wheyless cheese manufacturing could be performed anywhere, irrespective of the local availability of cow’s milk.

## Data Availability

The data presented in this study are available on request from the corresponding author.
